# Returning value from the *All of Us* Research Program to PhD-level nursing students using ChatGPT as programming support: results from a mixed-methods experimental feasibility study

**DOI:** 10.1093/jamia/ocae208

**Published:** 2024-07-29

**Authors:** Meghan Reading Turchioe, Sergey Kisselev, Ruilin Fan, Suzanne Bakken

**Affiliations:** School of Nursing, Columbia University, New York, NY 10032, United States; School of Nursing, Columbia University, New York, NY 10032, United States; Graduate School of Arts and Sciences, Columbia University, New York, NY 10027, United States; School of Nursing, Columbia University, New York, NY 10032, United States; Department of Biomedical Informatics, Columbia University, New York, NY 10032, United States; Data Science Institute, Columbia University, New York, NY 10027, United States

**Keywords:** AI (artificial intelligence), nursing informatics, informatics, medical informatics, nursing students, graduate nursing education, nursing education research

## Abstract

**Objective:**

We aimed to evaluate the feasibility of using ChatGPT as programming support for nursing PhD students conducting analyses using the *All of Us* Researcher Workbench.

**Materials and Methods:**

9 students in a PhD-level nursing course were prospectively randomized into 2 groups who used ChatGPT for programming support on alternating assignments in the workbench. Students reported completion time, confidence, and qualitative reflections on barriers, resources used, and the learning process.

**Results:**

The median completion time was shorter for novices and certain assignments using ChatGPT. In qualitative reflections, students reported ChatGPT helped generate and troubleshoot code and facilitated learning but was occasionally inaccurate.

**Discussion:**

ChatGPT provided cognitive scaffolding that enabled students to move toward complex programming tasks using the *All of Us* Researcher Workbench but should be used in combination with other resources.

**Conclusion:**

Our findings support the feasibility of using ChatGPT to help PhD nursing students use the *All of Us* Researcher Workbench to pursue novel research directions.

## Background and significance

The *All of Us* Research Program supported by the National Institutes of Health (NIH) is a large, innovative, and rich dataset that is well suited for use by biomedical researchers including nurse scientists to answer pressing research questions. Numerous sources of data collected through the program include surveys, clinical assessments, wearables, electronic health records, and genomic data from over 750 000 participants to date, with an ultimate target of one million participants.^1^ The *All of Us* Researcher Workbench is a secure data enclave in which users can explore and analyze these data.

Community engagement is a defining feature of the *All of Us* Research Program. Nurses are key partners in community-based research; the Future of Nursing Report identifies the key role of nurses in communities to advance health equity.^2^ Additionally, the National Institute of Nursing Research (NINR) Strategic Plan focuses heavily on the community as a core focus of current nursing research.^3^ Thus, there is a need for strategies that raise awareness and build nurse researcher competencies in the use of the *All of Us* Researcher Workbench.

However, nurses typically have limited exposure to programming and may report low self-efficacy and high frustration when they encounter programming tasks and concepts.^4^ They therefore are likely to face challenges utilizing the Researcher Workbench to develop and pursue scientific research questions. Additionally, PhD-prepared nurses need a conceptual and applied technical foundation to be able to collaborate productively with experts in data science on scientific projects.^5^ Tools to facilitate learning programming for PhD-level nursing students may help achieve these important competencies.

Generative artificial intelligence (AI) tools are rapidly being adopted. For example, the general AI chatbot, ChatGPT, has 100 million weekly users.^6^ Within informatics research and education, there is exciting potential to use generative AI tools to mitigate the high cognitive load associated with learning programming as a novice.^7^ Therefore, in this study we hypothesized that tools that lower the cognitive load associated with programming by providing temporary support through guidance and examples (eg, “cognitive scaffolding”), such as ChatGPT which can assist with writing and debugging code, will be an important strategy in PhD nursing education. The objective of this study was to evaluate the feasibility of using ChatGPT for programming support for PhD-level nursing students.

## Methods

### Study design

We employed an experimental feasibility study design within a data science and visualization course that is required for all second-year PhD-level nursing students. The course involves a laboratory component in which students complete programming assignments using the R programming language and Jupyter notebook to complement didactic content.

For the assignments, students first select a research question of interest and create a study cohort in the *All of Us* Researcher Workbench. They then complete 6 programming assignments that, together, progressively answer their research question. These include conducting descriptive statistics on key variables, using data visualizations to further examine the variables and associations of interest, developing a statistical analysis plan, conducting and interpreting bivariate regression models between the primary independent and dependent variables, conducting and interpreting multivariate regression models adjusting for important confounding variables, and completing and visualizing the final multivariate analysis. Assignments were graded based on code that ran without syntax errors and accomplished its objectives.

**Figure 1.**
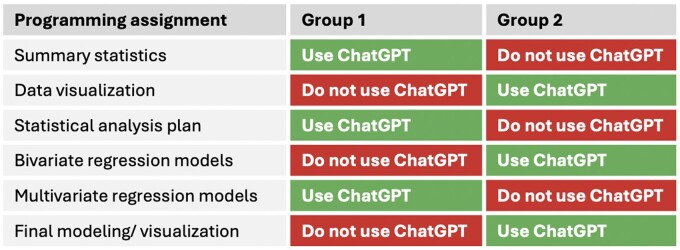
Group-level randomization of ChatGPT.

In this study, students were prospectively randomized to 1 of 2 groups for the semester. Each group could use ChatGPT for programming support on alternating assignments (Figure 1). This design was used to allow us to isolate ChatGPT’s effect from the difficulty of the specific assignment and create fairness so that all students had equal opportunities to use ChatGPT for assistance. All students received a subscription to ChatGPT 4.0 for the duration of the class at no cost. For all assignments, including those for which a group was asked not to use ChatGPT, students were permitted to use other outside resources (web searches, notes from other courses, Instructor support).

The Columbia University IRB approved this study. Students were permitted to opt out of the data being used for this research study if they did not consent to participate.

### Conceptual underpinning

**Figure 2.**
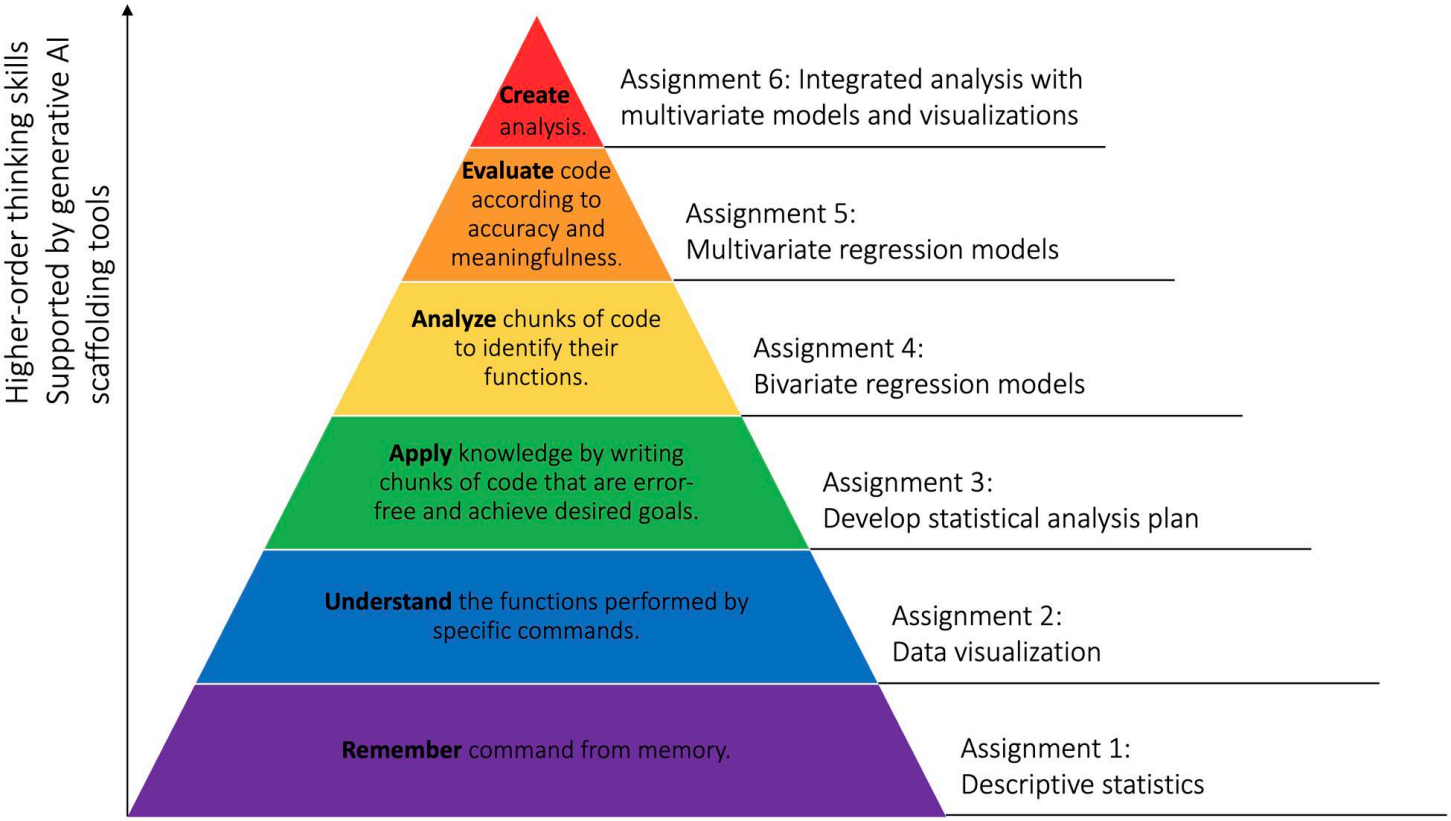
Bloom’s taxonomy of learning adapted to this study.

This study was grounded in Bloom’s Taxonomy of Learning, which orders cognitive skills by complexity (Figure 2).^8^ Specifically, we sought to investigate whether ChatGPT would provide the cognitive scaffolding needed to help students engage in the higher-order thinking and cognitive skills at the top of the taxonomy (analyze, evaluate, and create code), rather than remaining in lower-order thinking (remember and understand code). Each subsequent programming assignment tested a new level of the taxonomy such that the last assignments required higher-order thinking. We assessed whether ChatGPT would help all students progress to higher-order thinking through the accurate completion of all 6 assignments. The pedagogical framework and aims were developed and refined in collaboration with the Columbia Center for Teaching and Learning.

### Quantitative data collection and analysis

For each assignment, students also self-reported the amount of time it took to complete the assignment in minutes and rated their confidence in the correctness of their assignment on a scale of 1-5 (5 being the most confident). Quantitative data were analyzed using basic descriptive statistics to summarize responses and examine differences in completion time and confidence by assignment and by self-reported data science competency (including both programming and statistical/analytic skills) at the start of the course. We computed the median and interquartile range because of the presence of an outlier student whose assignment completion time was notably higher than other students on all assignments (regardless of whether ChatGPT was used).

### Qualitative data collection and analysis

For each of the 6 programming assignments, students wrote reflections of approximately 200 words each (for a total of approximately 1200 words throughout the study) addressing 4 topics: (1) barriers they faced that forced them to turn to an outside resource (ie, online resources, instructor support, and ChatGPT when permitted), (2) whether the resources provided accurate and helpful answers, (3) what they learned by consulting the resources, and (4) whether and how they modified the way they used the resources over time. Qualitative data were analyzed using directed content analysis. The 4 reflection prompts served as the initial codebook and new themes that emerged were incorporated. A Teaching Assessment Fellow at the Columbia Center for Teaching and Learning (R.F.) conducted the initial analysis and a second researcher (M.R.T.) periodically reviewed the coding scheme and audit trail. They discussed differences in interpretation until they agreed upon a final coding scheme.

## Results

Ten students enrolled in the course and all consented to the research study, but 1 student auditing the course did not submit data, resulting in a sample size of 9. At the start of the course, 4 students self-identified as a “novice” in their data science competency, 4 as an “advanced beginner,” and 1 as “proficient.”

### Assignment completion time

Assignment completion times are shown in Table 1. All students completed all of the assignments with full credit, indicating that the assignments were completed without syntax errors and achieved the desired goal. Across all assignments and groups, the median completion time was the same for both assignments when ChatGPT was used (median 120 minutes, interquartile range [IQR] 45-180) and when it was not (median 120 minutes, IQR 45-195). For students who identified as a “novice” in data science at the start of the course, the median completion time was shorter when using ChatGPT (median 85 minutes, IQR 45-210) versus without it (median 120 minutes, IQR 56-173) but not those who identified as an “advanced beginner” or “proficient.” ChatGPT saved time for groups who used it on assignments focused on planning and executing bivariate regression models but not for descriptive statistics or multivariate regression models.

**Table 1.** Differences in assignment completion time and self-reported confidence in the correctness of assignment between weeks students were permitted to use ChatGPT versus not permitted to use it (median [IQR] minutes), *n* = 9.

	Completion time			Confidence^a^		
	Overall	GPT	No GPT	Overall	GPT	No GPT
Across all assignments/students	120 (45-180)	120 (45-180)	120 (45-195)	3.0 (2.5-4.0)	3.0 (2.5-4.0)	3.0 (2.5-4.0)
By data science competency						
Novice (*n* = 4)	120 (45-195)	85 (45-210)	120 (56-173)	3.0 (3.0-4.0)	3.0 (3.0-4.0)	3.0 (3.0-4.0)
Advanced beginner (*n* = 4)	120 (41-248)	120 (53-255)	105 (41-248)	3.0 (2.0-3.3)	3.0 (2.0-3.3)	3.0 (2.0-3.3)
Proficient (*n* = 1)	60 (49-60)	60 (53-60)	60 (53-70)	4.0 (3.3-4.0)	4.0 (4.0-4.0)	3.0 (3.0-3.5)
By task/assignment						
Descriptive statistics	120 (60-180)	120 (60-180)	115 (68-173)	3.0 (2.0-3.0)	3.0 (2.0-3.0)	2.5 (2.0-3.0)
Data visualization	60 (45-180)	120 (56-240)	60 (45-60)	3.0 (2.0-3.0)	3.0 (2.8-3.3)	3.0 (2.0-3.0)
Develop statistical analysis plan	120 (30-210)	30 (30-120)	165 (101-233)	3.0 (2.0-3.0)	3.0 (2.0-3.0)	3.0 (2.8-3.3)
Bivariate regression models	120 (60-180)	90 (56-165)	120 (90-180)	3.0 (2.0-3.0)	2.5 (1.8-3.3)	3.0 (3.0-3.0)
Multivariate regression models and visualizations	120 (60-180)	120 (30-180)	120 (105-195)	4.0 (3.0-4.0)	4.0 (4.0-4.0)	3.0 (2.8-3.3)
Multivariate regression models and visualizations	45 (45-165)	83 (45-165)	45 (40-120)	4.0 (3.0-4.0)	3.5 (3.0-4.0)	4.0 (4.0-4.0)

a Confidence scores ranged from 1 to 5, with 5 representing the highest confidence.

### Confidence in task accuracy

Confidence scores are shown in Table 1. Across all assignments and groups, the median confidence score was 3.0 for both assignments when ChatGPT was used and when it was not. Confidence was higher for the student who self-reported being proficient in data science at the start of the course when they were using ChatGPT versus not, and higher for assignments related to running and interpreting multivariate regression models when students were using ChatGPT versus not.

### Qualitative reflections

A summary of qualitative themes and illustrative quotes are shown in Table 2, with additional sub-themes and quotes provided in Table S1. Students reported technical and conceptual barriers to completing the analysis and specific issues with the Researcher Workbench which caused them to seek outside help. Many reported using ChatGPT as a “first step” on the weeks it was permitted, and used with other resources. Students reported that ChatGPT helped generate and troubleshoot code and saved time, although it was occasionally unhelpful or inaccurate. Students found other online resources, class notes, and instructor support helpful, mostly for conceptual guidance. Regarding the learning process, students reflected that ChatGPT supports learning through examples and troubleshooting, whereas other resources bolster conceptual knowledge. Students improved their prompt engineering skills over time to use ChatGPT more effectively; for example, they learned that prompts that included their code and error messages verbatim paired with more specific questions elicited more helpful responses from ChatGPT compared to more general conceptual questions. Similarly, over time they also learned how to better craft search strategies in online search engines.

**Table 2.** Qualitative themes from students’ weekly reflections on programming assignments.

Theme	Summary	Illustrative quotes
Barriers causing students to seek help	Students reported technical and conceptual barriers to completing the analysis and specific issues with the data enclave.	“The apprehension of making errors or encountering unexpected issues in R Studio triggers a sense of panic in me.” “I have found that the *All of Us* notebook is particularly frustrating to use, so I write and make comments in an R notebook, then copy into the *All of Us* environment.”
Selecting resources	ChatGPT was selected as a “first step” and used with other resources.	“Having this premature engagement with [ChatGPT] before modeling is helpful.”
Using ChatGPT for specific tasks	ChatGPT helped students generate and troubleshoot code, saved time, and reduced frustration. It was occasionally unhelpful or inaccurate.	“The ability to easily copy and paste not only my code but my errors and warning messages has been very helpful to move the data wrangling process along. I still have to check to make sure the codes ChatGPT provides do not result in the loss of any of my data, but it at least allows me to move forward with analyses. Indeed, I have gotten a lot further this week than last week largely due to being able to use ChatGPT.” “I used ChatGPT to get the code for a multivariable linear regression model. This saved me a lot of time as the other weeks without ChatGPT I had a hard time being able to run my analysis.”
Using other resources for specific tasks	Students found other online resources, class notes, and instructor support helpful, mostly for conceptual guidance.	“ChatGPT transformed the data with different labels. This required a consultation with the instructor to ensure I am not accidentally losing data.”
The process of learning and skill-building	ChatGPT supports learning through examples and troubleshooting. Other resources bolster conceptual knowledge.	“For the previous assignment on summary characteristics, ChatGPT did teach me how to create tables. I carried that knowledge into this week.”
Refining use with experience	Students improved their prompt engineering skills over time to use ChatGPT more effectively.	“I would try to explain my error, but I found copying and pasting not the error message and initial code is most helpful in getting an accurate output.”

## Discussion

In this mixed-methods experimental feasibility study, ChatGPT helped students write and troubleshoot code, reduced frustration, and saved time spent on assignments for novices (median 35-minute difference in completion time for novices when ChatGPT was used). Students were able to progress further and complete more advanced statistical analyses in the Researcher Workbench than in prior years without the use of ChatGPT. ChatGPT appeared to save the most time for students who rated themselves as “novices” in data science and for tasks involving developing a statistical analysis plan and building bivariate regression models. Importantly, ChatGPT occasionally offered incorrect guidance and students therefore concluded that it should be used in conjunction with other resources, rather than as their only resource. Moreover, they noted that certain questions pertaining specifically to the *All of Us* Research Program were better answered on its website directly, as ChatGPT was not familiar enough with the program to answer specific questions about it.

The central question of this study was whether ChatGPT would facilitate higher-order conceptual thinking or whether, conversely, it would short-circuit learning by providing superficial technical support without the crucial conceptual foundations. Students reported learning the tasks’ conceptual underpinnings by leveraging ChatGPT with other resources and applying these foundations to subsequent assignments. Moreover, ChatGPT helped all students complete all assignments, including the final ones that required the highest-order thinking. The qualitative findings suggest that the most important function of ChatGPT was to help students overcome the time-consuming and frustrating barriers and bugs in programming that might otherwise significantly hinder their ability to progress in their analyses. This was particularly noticeable for students who were “novices” in data science, for whom the examples and troubleshooting support provided by ChatGPT were most helpful. There may be a ceiling effect, such that ChatGPT is less helpful for more advanced students; this warrants more exploration in future work.

Similarly, prior work has demonstrated that scaffolding tools ease the cognitive load when novice students are learning programming in other disciplines.^7^ Although ChatGPT has been recognized as a new scaffolding tool,^9^ there has been little empirical research to date confirming its utility. It is important to note that the effectiveness of AI-based tools may vary by students’ proficiency in prompt engineering, which we did not provide formal education on but which may be important in future work.^10^ Additionally, in this study some students were more comfortable engaging with ChatGPT in a language other than English, which we observed could produce different output since the underlying large language model is aligned differently in different languages.^11^

This study is limited by a small sample size tied to enrollment in the course, limiting our ability to conduct inferential statistics or to extrapolate our findings broadly. Additionally, our counterbalancing design created fairness among students, but made it more difficult to disentangle the impact of ChatGPT, particularly when skills were transferred over time, compared to a two-arm randomization scheme without crossover. Future research replicating this study in other settings, formally assessing the potential influence of ChatGPT on logic errors (as opposed to syntax only), and quantifying changes in cognitive load related to ChatGPT (for example, using the NASA Task Load Index method^12^) will be valuable in describing the potential for ChatGPT to help return value from the *All of Us* Research Program to nurse scientists and other important communities.

## Conclusion

This feasibility study provides evidence that the use of ChatGPT allowed PhD-level nursing students to move toward higher-order conceptual knowledge needed to manipulate datasets and variables in the *All of Us* Researcher Workbench. Therefore, demonstrating the feasibility of using ChatGPT as an effective scaffolding tool in conjunction with other resources for teaching data science and visualization. As AI tools to support data science work advance, so will the capabilities of nurses, researchers, and other communities using powerful datasets such as the *All of Us* Researcher Workbench to pursue novel research directions.

## Supplementary Material

ocae208_Supplementary_Data

## Data Availability

Given the small sample and the qualitative nature of the reflection data, the data are not available for sharing.
