# Supplementary material for: Returning value from the All of Us Research Program to PhD-level nursing students using ChatGPT as programming support: results from a mixed-methods experimental feasibility study
Source: J Am Med Inform Assoc. 2024 Jul 29;31(12):2974–9. doi: 10.1093/jamia/ocae208 (PMC11631052; doi:10.1093/jamia/ocae208)
Supplement: ocae208_Supplementary_Data [file ocae208_supplementary_data.docx]

| **Supplementary Table 1.** Qualitative themes and illustrative quotes from students’ reflections (n=9) | | |
| --- | --- | --- |
| **Sub-theme** | **Summary** | **Illustrative quote** |
| **Theme 1: Barriers causing students to seek help** | | |
| 1.1 Lack of programming background | Students’ lack of familiarity with programming in R and Jupyter notebook created barriers from the outset. | “I've been facing challenges in leveraging R for my analysis. Despite dedicating time to acquaint myself with R Studio, I realize there's a need for further practice to gain proficiency. Gradually, I'm becoming more comfortable with the R Studio environment, yet the apprehension of making errors or encountering unexpected issues in R Studio still triggers a sense of panic in me.” (Student 9, Lab 8, ChatGPT not permitted) |
| 1.2 Difficulty merging and cleaning datasets | Students had difficulty merging and cleaning datasets with different dimensions and manipulating different types of variables. | “I was initially confused on why I could not merge the dataframes … I was also confused on what type of summary statistics I could do, since the survey items are mixed in the same column.” (Student 5, Lab 5, ChatGPT permitted) |
| 1.3 Difficulty conducting statistical analyses | Students needed support with both the conceptual and technical aspects of conducting regression analyses and other statistical analyses and creating data visualizations. | “My challenges this week related to my fundamental statistical skills and minimal ability to handle skewed data. Despite referring to notes from previous regression courses to learn how to transform the data proved challenging.” (Student 4, Lab 9, ChatGPT not permitted) |
| 1.4 Challenges specific to the “All of Us” platform | Students reported three main challenges: (1) extremely small subsamples of cohorts of interest, (2) confusion with similar variable names across datasets, and (3) technical issues such as the kernel repeatedly dying. | “Calculating the duration of moderate and vigorous physical activity (PA) proved more complex than anticipated. Some questions shared the same codes/names, which were mixed, leading to confusion. Second, after cleaning the data by removing participants who skipped or left PA questions unanswered, only 18 participants [in my cohort] remained. To avoid any possibility of identifying participants, I need a sample size larger than 20 participants.” (Student 7, Lab 8, ChatGPT permitted) |
| **Theme 2: Rationale for selecting specific resources** | | |
| 2.1 Deciding to use ChatGPT | Most students opted to use ChatGPT (when permitted) as a first step because they lacked familiarity with a particular analysis or the R programming language. | “I used ChatGPT this week mostly in a premature manner to see its familiarity with multivariate analyses and suggestions for visualizations… having this premature engagement with the resource before modeling is helpful.” (Student 6, Lab 9, ChatGPT permitted) |
| 2.2 Missing ChatGPT when unavailable | Some students who were not permitted to use ChatGPT on a given week reported struggling without it. | “ChatGPT might have helped me troubleshoot my connection issues and error messages earlier.” (Student 5, Lab 6, ChatGPT not permitted) |
| 2.3 Deciding to use other sources when ChatGPT permitted | In a few cases, students opted to use other resources even when they were permitted to use ChatGPT because they sought information directly from the All of Us website. | “My kernel kept dying and so I went to google because I wanted resources from the actual All of Us website. After removing some columns in my tables, my kernel worked.” (Student 1, Lab 5, ChatGPT permitted) |
| **Theme 3: Using ChatGPT for specific tasks** | | |
| 3.1 Providing code | Most students used ChatGPT to generate code when getting started on a task such as merging datasets or running descriptive statistics. They reported it was often reliable and accurate, but not always. | “The ability to easily copy and paste not only my code but my errors and warning messages have been very helpful to move the data wrangling process along. I still have to check to make sure the codes chatGPT provides do not result in the loss of any of my data, but it at least allows me to move forward with analyses. Indeed, I have gotten a lot further this week than last week largely due to being able to use chatGPT.” (Student 5, Lab 7, ChatGPT permitted) |
| 3.2 Suggesting additional/ alternative approaches | ChatGPT occasionally provided helpful suggestions and advice beyond what the initial prompt requested. | “I used ChatGPT to help identify any additional analyses that I may not have considered. I asked ChatGPT to please create me a model with the listed binary variables for outcome and exposure including the provided covariates. Chat GPT also provided recommendations for model diagnostics, sensitivity analysis and reporting of the results as adjusted odds ratio.” (Student 2 , Lab 7, ChatGPT permitted) |
| 3.3 Efficiency and time management | Nearly all students perceived that ChatGPT saved time, created efficiencies, and helped overcome roadblocks. | “I only entered one prompt into chatGPT and I got the code I needed to run the analysis. This saved me a lot of time, as the other weeks without ChatGPT, I had a hard time being able to run my analysis.” (Student 1, Lab 9, ChatGPT permitted) |
| 3.4 Unhelpfulness/ inaccuracies | ChatGPT’s responses were sometimes inaccurate, lacked appropriate nuance for a particular dataset or statistical analysis, or were lengthy which caused some students to feel more overwhelmed. | “It wasn’t clear to me what ChatGPT was describing and I would get error messages when attempting to run its code. ChatGPT wasn’t incorrect in what it had recommended but that was a small quirk about Jupyter Notebook that wasn’t described.” (Student 2, Lab 5, ChatGPT permitted) |
| **Theme 4: Using other resources for specific tasks** | | |
| 4.1 Other online resources helpful for specific tasks | Students turned to a variety of online resources for support when ChatGPT was not permitted (and sometimes even when it was), including Google, Youtube, Stack Overflow, and the All of Us website. Overall, the resources were highly accurate and helpful in providing step-by-step tutorials on how to run specific code for a given task. | “Among the resources I've explored, YouTube's R tutorials have proven to be exceptionally beneficial, offering practical insights and step-by-step guidance. Google searches also provide a reliable fallback for quick solutions and additional information.” (Student 9, Lab 8, ChatGPT not permitted) |
| 4.2 Other online resources unhelpful for specific tasks | Students reported challenges troubleshooting coding errors using other resources, which was inefficient and time-consuming. | “I mainly directly search in Google's search engine. Then it navigated me to different coding forums [which] can provide helpful and correct answers to my questions, but it often takes a long time and trial and error.” (Student 8, Lab 5, ChatGPT not permitted) |
| 4.3 Instructor support | Nearly all students faced challenges that required consultation with an instructor; these primarily centered on technical issues (programs crashing due to large file sizes) and identifying subtle errors. Students expressed gratitude for instructor support as an option, especially when ChatGPT was not permitted. | “ChatGPT provided some code, but it transforms the data with different labels. This required a consultation with [the instructor] to ensure I am not accidentally losing data.” (Student 5, Lab 7, ChatGPT permitted) |
| 4.4 Current/ past course materials | Students used notes from prior statistics courses and code published in peer-reviewed papers, sometimes in conjunction with ChatGPT. | “This week’s lab included conducing multivariate analyses and I was part of the Chat GPT group. I actually started off this lecture by returning to some of my notes from a [regression] course I took over the summer because I wanted the refresher and to make connections between all I had been learning.” (Student 2, Lab 9, ChatGPT permitted) |
| **Theme 5: The process of learning and skill building** | | |
| 5.1 Learning with ChatGPT | ChatGPT mainly supported learning by providing examples and troubleshooting, which students applied in later work. Some students wondered if the quick answers provided by ChatGPT short-circuited the learning process. | “For the previous assignment, I was in the ChatGPT group and as I learned about summary characteristics the AI tool did teach me how to create tables. I carried that knowledge into this week [when I could not use ChatGPT]”. (Student 2, Lab 6, ChatGPT not permitted) |
| 5.2 Learning with other resources | Most students learned well with other resources, as well, and noted that they bolstered the conceptual knowledge needed to write accurate code. Some noted that inefficiencies in searching within other resources slowed learning. | “I learned from coding errors that a deep understanding of the statistical model in use is crucial. This is because the functions are quite flexible, and offer many parameters, which forced me to understand what they mean and how to adjust them based on the actual data, rather than use the default values.” (Student 8, Lab 7, ChatGPT not permitted) |
| **Theme 6: Refining use with experience** | | |
| 6.1 Refining use of ChatGPT | Students learned how to write more effective prompts over time which increased the effectiveness of ChatGPT. | “I have modified the way I use ChatGPT to get the most effective response. I originally would try to explain my error, but I found copying and pasting not only the error message but also the initial code, is most helpful in getting an accurate output from ChatGPT. I also realized ChatGPT retains memory with each prompt, so I do not have to explain the problem again with each question.” (Student 5, Lab 5, ChatGPT permitted) |
| 6.2 Refining use of other resources | Students needed to refine web search strategies to get the answer they needed and apply more energy to crafting a search and locating the information. | “When I am not in the ChatGPT group, this is when I need to be a bit more creative to find R code.” (Student 4, Lab 8, ChatGPT permitted) |
